# Ultrasonographic Evaluation of Nasal Tip Anatomy for Rhinoplasty Planning

**DOI:** 10.1055/a-2802-5847

**Published:** 2026-03-27

**Authors:** Chuong Dinh Nguyen, Ngoc Ba Nguyen, Hue Minh Ho, Anh Ngoc Hoang, Tung Thanh Nguyen, Quynh Ngoc-Thuy Ho, Tho Thi-Kieu Nguyen

**Affiliations:** 1 Department of Postgraduate Training, University of Medicine and Pharmacy at Ho Chi Minh City, Ho Chi Minh City, Vietnam; 2 Department of Plastic and Reconstructive Surgery, Gia Dinh People's Hospital, Ho Chi Minh City, Ho Chi Minh City, Vietnam; 3 Department of Diagnostic Imaging, Gia Dinh People's Hospital, Ho Chi Minh City, Vietnam; 4 Department of Otorhinolaryngology, Thuan An City Medical Center, Binh Duong, Vietnam; 5 Department of Otorhinolaryngology, University of Medicine and Pharmacy at Ho Chi Minh City, Ho Chi Minh City, Vietnam; 6 Department of Otorhinolaryngology, Pham Ngoc Thach University of Medicine, Ho Chi Minh City, Vietnam

**Keywords:** ultrasonography, nasal tip, Asian rhinoplasty, soft tissue envelope, septal cartilage, internal nasal valve angle

## Abstract

**Background:**

Rhinoplasty outcomes are heavily influenced by nasal tip anatomy, particularly the thickness of the soft tissue envelope (STE) and the underlying cartilaginous framework. Traditional assessment methods are subjective, prompting exploration of objective techniques such as ultrasonography. This study evaluates nasal tip anatomy using high-resolution ultrasound imaging and correlates quantitative findings with surgical implications.

**Methods:**

This prospective study enrolled 35 adult patients at a tertiary referral hospital. Patients with prior nasal surgery, injections, fractures, congenital anomalies, or significant skin disease were excluded. A 12-MHz ultrasound probe was used to measure STE thickness at the nasion, rhinion, and nasal tip, septal cartilage thickness and dorsal length, and the internal nasal valve angle (INVA).

**Results:**

STE thickness was greatest at the nasion (4.11 ± 0.51 mm), followed by the nasal tip (3.95 ± 0.54 mm), and thinnest at the rhinion (2.49 ± 0.16 mm). The mean septal cartilage thickness was 2.09 ± 0.30 mm at the premaxilla and thinner at the dorsum (1.54 ± 0.26 mm). The mean dorsal septal length was 22.8 mm. Male patients consistently exhibited thicker STEs and septal cartilage than female patients ( *p* < 0.001). The INVA averaged 25.7 degrees bilaterally.

**Conclusion:**

Ultrasonography provides a reliable, objective assessment of nasal anatomy for surgical planning. Recognition of variations in soft tissue and septal morphology facilitates tailored approaches, improving both aesthetic and functional outcomes, especially in patients with thick nasal skin.

## Introduction

Rhinoplasty, widely regarded as one of the most challenging aesthetic surgical procedures, demands precise anatomical knowledge and detailed preoperative planning to achieve predictable outcomes. Among the anatomical subunits, the nasal tip plays a pivotal role in determining nasal aesthetics but remains particularly challenging due to its complex framework, including the lower lateral cartilages, septum, bony skeleton, and the overlying soft tissue envelope (STE). Variations in these components significantly influence tip appearance across individuals and ethnic groups. 1 Skin thickness, in particular, profoundly affects surgical results: While thick skin may conceal minor cartilaginous imperfections, it often limits tip definition and increases the risk of postoperative dissatisfaction; conversely, thin skin may reveal underlying irregularities, compromising aesthetic harmony. 2 Asian populations generally present with thicker nasal skin and smaller quadrangular cartilage compared with their Caucasian counterparts. 3 4 These anatomical distinctions necessitate tailored surgical techniques, emphasizing the importance of individualized preoperative assessment.

However, accurate characterization of these anatomical variables requires reliable and objective assessment tools. Current clinical evaluations, primarily based on physical examination and palpation, are inherently subjective and prone to interobserver variability. Advanced imaging modalities like computed tomography (CT) and magnetic resonance imaging (MRI) provide more objective data but are not routinely used in aesthetic rhinoplasty due to cost, radiation exposure, and limited resolution for superficial structures. 3 These limitations highlight the demand for a safe, reproducible, and non-invasive imaging tool.

High-frequency ultrasonography offers several advantages in this regard. It enables precise, real-time measurement of both soft tissue and cartilaginous structures of the nasal tip without radiation exposure. 5 6 7 Recent advances in ultrasound technology have significantly improved image resolution, enabling precise delineation of STE structures and cartilaginous frameworks, which are critical for effective preoperative assessment and postoperative evaluation. 6 Although the feasibility of ultrasonography for nasal assessment has been demonstrated, its use remains underutilized, partly due to the lack of standardized protocols and limited clinical data.

The ultimate aim of this study is to establish prospective ultrasonographic reference values for nasal tip anatomy in an Asian cohort. By quantifying STE thickness at key nasal landmarks, characterizing septal cartilage morphology, and assessing internal nasal valve angles (INVAs), we sought to generate normative data that are directly applicable to clinical decision-making. Through these comprehensive assessments, this study positions ultrasonography as a reliable and clinically relevant adjunct to preoperative rhinoplasty planning, thereby supporting its integration into routine preoperative protocols to refine both aesthetic and functional outcomes in patients with distinctive anatomical features.

## Methods

### Study Design and Participants

This prospective, observational study was conducted at a tertiary referral hospital from October 2023 to September 2024. Ethical approval was obtained from the Institutional Review Board of the university and the hospital (1264 & 1265/HDDD-DHYD and 83 & 84/NDGD-HDDD). Written informed consent was obtained from all participants, including permission to use anonymized clinical photographs and ultrasound images for publication.

Patients eligible for the study included those aged 18 years and older who visited the plastic and reconstructive surgery department, without previous nasal surgical interventions, injections, fractures, congenital anomalies, or significant dermatological conditions. A total of 35 patients (20 males, 15 females) met these inclusion criteria. Demographic data, including age, sex, body mass index (BMI), and medical history, were recorded for each patient.

### Ultrasonographic Procedure

**Video 1** Standardized ultrasonographic protocol for rhinoplasty planning, demonstrating probe placement and representative screen recordings for nasal soft tissue thickness, septal cartilage measurements, and internal nasal valve angle.


All ultrasonographic imaging was performed by a single, experienced ultrasonographer, using a high-frequency 12 MHz linear probe (LOGIQ S7 Expert, GE Healthcare). Patients were positioned supine with their head stabilized in a neutral position. Standardized scans were acquired in both transverse and longitudinal planes, focusing on predefined anatomical landmarks: Nasion, rhinion, and nasal tip.

**Fig. 1:**
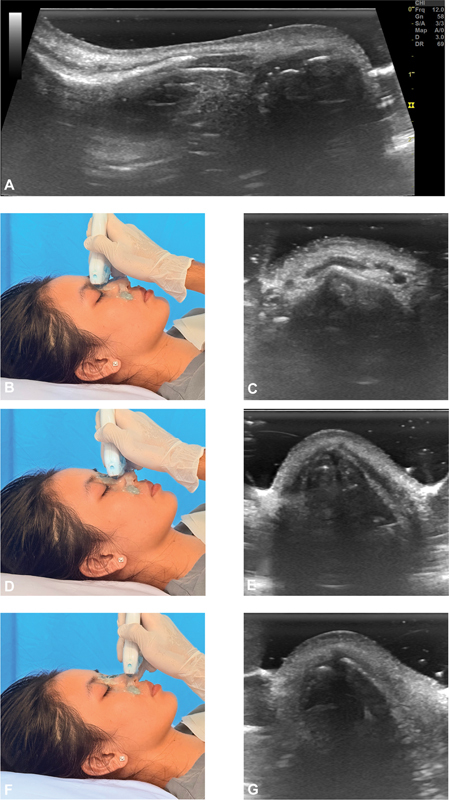
Ultrasound imaging of the nasal soft tissue envelope at three distinct nasal sites with corresponding probe positions. (A) Longitudinal scan along the nasal dorsum. (B, C) Axial scan and probe placement at the nasion. (D, E) Axial scan and probe placement at the rhinion. (F, G) Axial scan and probe placement at the nasal tip.

**Fig. 2:**
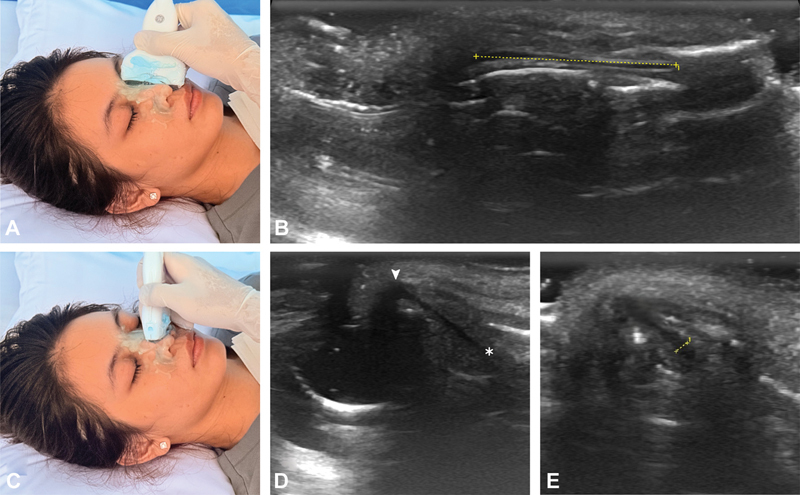
Ultrasound imaging of the nasal septal cartilage with illustrated probe positions. (A, B) The dorsal septal cartilage length (dashed line). (C–E) The caudal septal height from the premaxilla (asterisk) to the anterior septal angle (arrowhead). (D) Cartilage thickness at the premaxillary base (E).

Longitudinal scans, initiated at the nasion, extended caudally to the nasal tip. Transverse scans provided cross-sectional visualization of the nasal dorsum and its underlying structures at various points along its length ( Fig. 1 ). Slight sliding and tilting during the scan in a slightly oblique sagittal plane permitted an anterior–posterior axis view of the septal cartilage. Rotating the transducer in the lateral nasal cartilages area then provided views of the caudal nasal septum ( Fig. 2 ). The length of the cartilaginous septum at its anterior–superior border and the nasal length from nasion to nasal tip were measured at longitudinal views. Sagittal views facilitated landmark identification for subsequent axial measurements of STE thickness and the INVA.

**Fig. 3:**
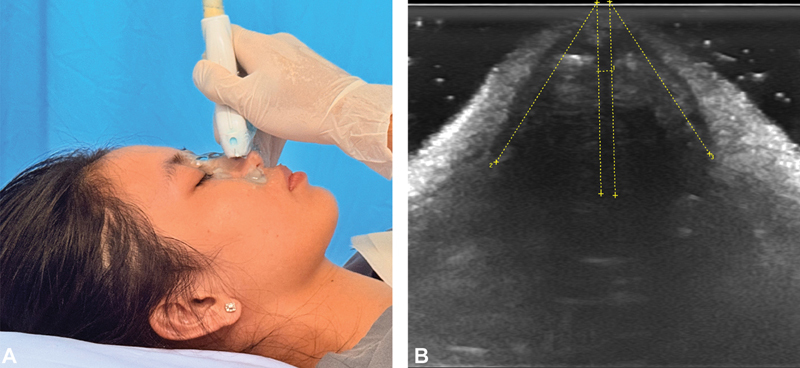
Ultrasound imaging of the internal nasal valve with illustrated probe position. ( **A** ) Probe placement. ( **B** ) Axial view showing internal nasal valve angle measurement.

STE thickness was defined as the distance from the periosteum or perichondrium to the epidermal surface. Septal cartilage thickness was measured on images obtained at the caudal nasal septum. To measure the INVA, the transducer was rotated to obtain an axial view at the caudal border of the alar cartilage, immediately superior to the lateral crus of the alar cartilage ( Fig. 3 ). The INVA, defined as the angle formed by the upper lateral cartilage and the nasal septum, was then measured bilaterally. All measurements were digitally recorded. To enhance clarity and reproducibility, annotated figures and supplementary ultrasound video clips are provided ( Video 1 ).

### Statistical Analysis

SPSS software version 22.0 (IBM SPSS) was used for data analysis. Descriptive statistics (means, standard deviations, medians, ranges) were calculated for all measured parameters. Comparative analyses were performed using Student's *t* -test for sex and age comparisons. Correlations between anatomical parameters were assessed using Pearson's correlation coefficient. Multivariable linear regression analysis was performed to identify independent predictors of the anatomical parameters, adjusting for age, sex, and BMI. Statistical significance was defined as *p* ≤ 0.05.

## Results

### Demographics

Thirty-five patients were included in the study. The mean age was 34.1 ± 12.7 years (range, 19–66; median 31 years). The mean height was 166.0 ± 7.8 cm, and the mean weight was 61.4 ± 12.5 kg, resulting in a mean BMI of 22.2 ± 2.9 kg/m ^2^ . For subgroup analyses, patients were stratified into two groups: ≤30 years ( *n* = 15) and >30 years ( *n* = 20). An overview of the measured values of the nose is given in Table 1 .

**Table 1:** Demographic characteristics and nasal measurements

Variable	Overall ( *n* = 35)	≤30 years ( *n* = 15)	>30 years ( *n* = 20)	*p* -Value
Age (years)	34.1 ± 12.7	23.4 ± 4.5	42.1 ± 10.8	
Height (cm)	166.0 ± 7.8	166.2 ± 10.6	165.2 ± 5.2	
Weight (kg)	61.4 ± 12.5	62.1 ± 15.7	60.9 ± 9.8	
BMI (kg/m ^2^ )	22.2 ± 2.9	22.2 ± 3.5	22.2 ± 2.5	
STE (mm)				
Nasion	4.11 ± 0.51	4.04 ± 0.56	4.17 ± 0.47	0.474
Rhinion	2.49 ± 0.16	2.49 ± 0.17	2.50 ± 0.16	0.818
Tip	3.95 ± 0.54	4.01 ± 0.71	3.90 ± 0.37	0.604
Septum				
Premaxilla thickness (mm)	2.09 ± 0.30	2.09 ± 0.28	2.09 ± 0.32	0.987
Dorsum thickness (mm)	1.54 ± 0.26	1.46 ± 0.27	1.60 ± 0.24	0.115
Dorsum length (mm)	22.81 ± 3.70	22.09 ± 3.66	23.34 ± 3.74	0.326
INVA (degrees)				
Left	25.7 ± 4.3	27.1 ± 5.0	24.7 ± 3.5	0.127
Right	25.8 ± 4.4	27.2 ± 5.1	24.7 ± 3.4	0.111

Abbreviations: BMI, body mass index; INVA, internal nasal valve angle; STE, soft tissue envelope.

Values are presented as mean ± standard deviation. *p* -Values were calculated using a *t* -test.

### Soft Tissue Envelope Thickness

**Fig. 4:**
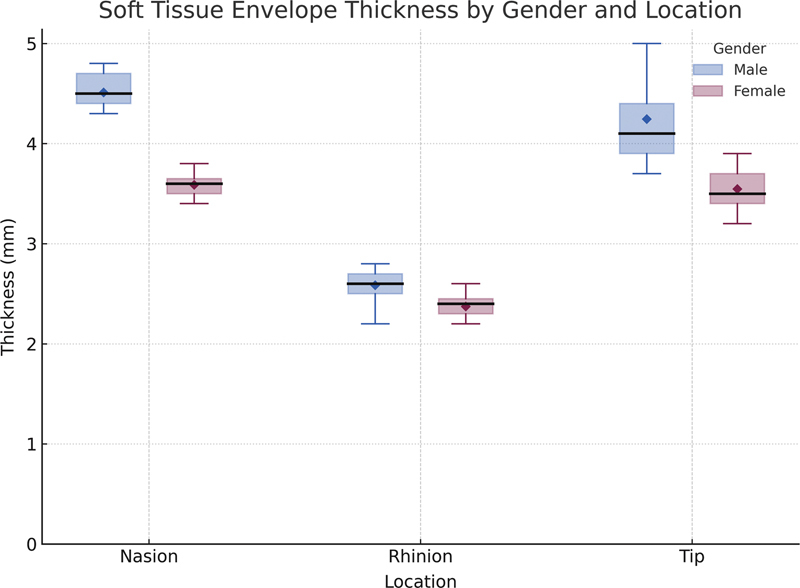
Boxplot showing soft tissue envelope thickness at three nasal landmarks (nasion, rhinion, and tip), stratified by gender.

The mean thickness of the STE was 4.11 ± 0.51 mm at the nasion, 2.49 ± 0.16 mm at the rhinion, and 3.95 ± 0.54 mm at the nasal tip. No statistically significant differences were found between the ≤30 and >30 groups (nasion: 4.04 ± 0.56 vs. 4.17 ± 0.47 mm, *p* = 0.47; rhinion: 2.49 ± 0.17 vs. 2.50 ± 0.16 mm, *p* = 0.82; tip: 4.01 ± 0.71 vs. 3.90 ± 0.37 mm, *p* = 0.60). Male patients consistently exhibited thicker STE than females at all sites, independent of age (nasion: +0.92 mm, *p* < 0.001; tip: +0.70 mm, *p* < 0.001; rhinion: +0.21 mm, *p* < 0.001; Fig. 4 ).

### Septal Cartilage Measurements

The mean septal cartilage thickness was 2.09 ± 0.30 mm at the premaxilla and 1.54 ± 0.26 mm at the anterior–superior dorsum. The mean length of the dorsal cartilaginous septum was 22.81 ± 3.70 mm. Between age groups, no significant differences were found in septal thickness (premaxilla: 2.09 ± 0.28 vs. 2.09 ± 0.32 mm, *p* = 0.99; dorsum: 1.46 ± 0.27 vs. 1.60 ± 0.24 mm, *p* = 0.12). Septal length was slightly greater in the >30 group than in the ≤30 group (23.34 ± 3.74 vs. 22.09 ± 3.66 mm), though this difference was not statistically significant ( *p* = 0.33). Males demonstrated significantly greater septal thickness and length than females (thickness at premaxilla: +0.33 mm, *p* < 0.001; thickness at dorsum: +0.24 mm, *p* = 0.006; length: +4.07 mm, *p* < 0.001).

### Correlation Analyses (Soft Tissue Envelope and Septal Thickness)

Correlation analysis demonstrated a modest, non-significant association between soft tissue thickness at the nasal tip and septal thickness at the premaxilla (Pearson's *r* = 0.215; *p* = 0.214). A significant correlation was found between soft tissue thickness at the rhinion and cartilage thickness at the anterior–superior nasal dorsum (Pearson's *r* = 0.374; *p* = 0.027).

### Internal Nasal Valve Angle

The INVA measured 25.7 ± 4.3 degrees on the left side and 25.8 ± 4.4 degrees on the right side (range, 17.8–34.0 degrees). Age showed a negative correlation with INVA (left: *r* = −0.33, *p* = 0.053; right: *r* = −0.338, *p* = 0.047). Group comparisons showed wider angles in the ≤30 group than in the >30 group (left: 27.1 ± 5.0 degrees vs. 24.7 ± 3.5 degrees, *p* = 0.13; right: 27.2 ± 5.1 degrees vs. 24.7 ± 3.4 degrees, *p* = 0.11), although these differences were not statistically significant.

### Multivariable Regression Analysis

As summarized in Table 2 , male sex was the strongest independent predictor of STE thickness at the nasion ( *p* < 0.001), rhinion ( *p* = 0.003), and nasal tip ( *p* < 0.001), as well as septal base thickness ( *p* < 0.001). Interestingly, BMI showed a significant positive association with dorsal septal cartilage length ( *p* = 0.019) but did not significantly influence skin thickness. Furthermore, age was found to be a significant negative predictor of the mean INVA ( *p* = 0.011).

**Table 2:** Multivariable linear regression analysis of anatomical parameters

Dependent variables	Model *R* ^2^	Age ( *p* -value)	Sex ( *p* -value)	BMI ( *p* -value)
STE				
Nasion	0.854	0.486	<0.001	0.072
Rhinion	0.451	0.757	0.003	0.205
Tip	0.445	0.478	<0.001	0.513
Septum				
Premaxilla thickness	0.423	0.234	<0.001	0.052
Dorsum length	0.462	0.169	0.053	0.019
Mean INVA	0.514	0.011	<0.001	0.407

Abbreviations: BMI, body mass index; INVA, internal nasal valve angle; STE, soft tissue envelope.

## Discussion

Rhinoplasty is recognized as one of the most challenging surgical procedures in facial plastic surgery, due to the inherent complexity and variability of nasal anatomy. The nasal tip, representing the most visually prominent and anatomically intricate component of the nasal structure, significantly influences both aesthetic outcomes and patient satisfaction. Precise preoperative evaluation of the nasal tip, including assessment of STE thickness and the underlying cartilaginous framework, is pivotal to achieving predictable postoperative results. Because these structural elements vary considerably among individuals and ethnic groups, 1 a reliable understanding of their morphology is essential for tailoring surgical strategies. In this context, the present study provides ultrasonographic data on STE thickness, septal cartilage morphology, and INVAs in an Asian cohort. These findings provide normative reference values and clarify how ultrasonography can inform surgical planning and improve both aesthetic and functional outcomes.

In our study, STE thickness varied significantly across nasal subunits, with the greatest thickness found at the nasion region (4.11 ± 0.51 mm), followed closely by the nasal tip (3.95 ± 0.54 mm), and the thinnest area at the rhinion (2.49 ± 0.16 mm). These findings are within previously reported ranges for Caucasian and Asian cohorts, suggesting consistent patterns of nasal soft tissue distribution across different ethnic groups. 3 8 Furthermore, our results indicate notable gender differences in STE thickness, with male subjects consistently exhibiting thicker nasal skin across all measured regions than female subjects, highlighting the importance of individualized surgical planning. Importantly, our multivariable analysis confirmed that male sex predicts thicker STE even after controlling for BMI ( Table 2 ). This indicates that the difference is driven by biological sex rather than body weight. Our mean tip thickness (3.95 mm) aligns with Chen et al 3 and exceeds values reported in Caucasian cohorts (2.3–3.1 mm), 8 9 10 a difference with practical implications for tip definition and the risk of supratip deformity. Thick nasal skin presents a unique surgical challenge. Although it can effectively conceal minor irregularities in the underlying cartilaginous structure, excessive thickness significantly limits nasal tip definition and increases the risk of postoperative supratip deformity. 11 Previous studies by Cho et al have shown that STE thickness exceeding approximately 3.4 mm at the tip correlates negatively with aesthetic outcomes. 12 In our patient cohort, the average nasal tip thickness was 3.95 mm, slightly higher than Cho et al's threshold, underscoring the need for deliberate preoperative planning in thick-skinned patients.

Septal cartilage dimensions also demonstrated predictable patterns. We found the premaxillary septum to be thicker (2.09 ± 0.30 mm) than the dorsal portion (1.54 ± 0.26 mm), closely reflecting cadaveric data from Hwang et al, 13 who reported base thickness of 2.19 mm and dorsum thickness of 1.03 to 1.50 mm. The mean dorsal length of the cartilaginous septum in our cohort was 22.8 mm, a value consistent with previously reported dorsal dimensions in Asian and Caucasian populations. Although this is shorter than the overall mean septal cartilage length (33.1 mm) described in cadaveric studies, 14 15 16 17 it nevertheless remains clinically sufficient for routine grafting. In many Asian patients, reliable tip support may therefore require septal extension grafts of approximately 25 mm. 18 We found no significant differences related to age. This finding is consistent with reports that septal cartilage growth increases after birth and continues into the twenties before stabilizing. 16 In contrast, male patients demonstrated greater septal thickness and length, reinforcing sex as a determinant of graft availability.

The INVA in our cohort was measured at approximately 25 to 26 degrees bilaterally. These results are comparable to CT-based studies in Asian populations (21.6 ± 4.5 degrees in South Koreans, 19 20.48 ± 2.99 degrees in Chinese patients 20 ) and somewhat broader than those reported in Caucasian cohorts. 21 22 23 MRI studies, such as by San Nicoló et al, demonstrated similar values (mean 28.6 degrees). 24 These comparisons suggest that our ultrasonographic measurements are physiologically plausible and align more closely with CT and MRI data from Asian cohorts. Regarding aging, while categorical comparison between age groups (≤30 vs. >30 years) showed no significant difference, our regression analysis revealed a subtle but significant inverse association between age and INVA ( *p* = 0.011). This indicates that anatomical narrowing may occur progressively, although this structural change does not necessarily compromise functional airway patency, which remains stable across adulthood as reported by Wang et al. 25

In the context of existing literature, our findings demonstrate close agreement with CT, MRI, and cadaveric studies: STE values correspond with both Asian and Caucasian cohorts, septal cartilage dimensions match prior radiologic and morphometric analyses, and INVA measurements align with advanced imaging. This consistency supports the validity of ultrasonography as a reliable adjunct for rhinoplasty planning, as summarized in Table 3 .

**Table 3:** Comparative measurements from the literature

Study (year)	Modality	Population	Measurement 1	Measurement 2	Measurement 3
A. Soft tissue envelope (mm)			Nasion or Radix	Rhinion	Nasal tip
Our study	Ultrasound	Vietnamese	4.11 ± 0.51	2.49 ± 0.16	3.95 ± 0.54
Chen et al, 2024 3	Ultrasound	Chinese	4.13 ± 0.72	2.25 ± 0.51	4.07 ± 0.72
Assiri et al, 2023 32	Ultrasound	Middle Eastern	4.04 ± 1.01	1.81 ± 0.70	3.17 ± 0.71
Cho et al, 2011 12	CT	Korean	3.3 ± 1.3	2.4 ± 1.0	2.9 ± 0.6
Charoenlux and Supanakorn, 2024 33	CT	Thai	2.77 ± 0.71	1.75 ± 0.63	3.23 ± 0.91
Dey et al, 2019 8	CT	Caucasian	6.7 ± 1.7 a	2.1 ± 0.7	3.1 ± 0.6
Eggerstedt et al, 2020 9	CT	Mixed	5.28 ± 1.90 a	1.57 ± 0.88	2.70 ± 0.79
Eggerstedt et al, 2020 9	CT	Asian	4.43 ± 1.63 a	1.34 ± 0.81	2.75 ± 0.71
Jomah et al, 2019 34	CT	Middle Eastern	3.96 ± 1.08	1.86 ± 0.62	3.32 ± 0.78
Alharethy et al, 2018 35	CT	Middle Eastern	3.89 ± 1.48	1.16 ± 0.60	2.93 ± 0.97
Çavuş Özkan et al, 2020 36	MRI	Turkish	6.22 ± 1.51	1.81 ± 0.30	2.67
**B. Septal parameters (mm)**			**Dorsal length**	**Base thickness**	**Dorsal thickness**
Our study	Ultrasound	Vietnamese	22.81 ± 3.70	2.09 ± 0.30	1.54 ± 0.26
Han et al, 2019 15	Cadaver	Chinese	25.63 ± 4.27		
Pernia et al, 2011 17	Cadaver	Filipino	22.3		
Godley, 1997 14	Cadaver	Caucasian	21 ± 5		
Simon et al, 2013 37	Cadaver	Caucasian	27.1 ± 4.30		
Kim et al, 2008 16	MRI	Korean	26 ± 4		
Kim and Yang, 2019 38	CT	Korean	27 ± 2.9		
Eid et al, 2021 39	Ultrasound	Caucasian		1 (overall)	
Stenner et al, 2017 6	Ultrasound	Caucasian		1.2 ± 0.05	
Hwang et al, 2010 13	Cadaver	Korean		2.19 to 3.03	1.03 to 1.50
Samibut et al, 2021 40	Cadaver	Thai		0.87 ± 0.21	1 ± 0.4
De Pochat et al, 2011 41	Cadaver	Brazilian		1.28 ± 0.46	1.04 ± 0.39
Mowlavi et al, 2006 42	Cadaver	Caucasian		2.7 ± 0.1	1.2 ± 0.1
Saunders et al, 1995 43	Cadaver, MRI	Caucasian		1.3	2.2 ± 0.42
**C. Internal nasal valve angle (degrees)**			**Left (or mean; degrees)**	**Right (degrees)**	**Note**
Our study	Ultrasound	Vietnamese	25.7 ± 4.3	25.8 ± 4.4	Bilateral
Suh et al, 2008 19	CT	Korean	21.6 ± 4.5		Mean
Suh et al, 2008 19	Endoscopy	Korean	19.3 v 3.6		Mean
Englhard et al, 2016 23	CT	Asian	21.8 ± 2.9		Mean
Englhard et al, 2016 23	CT	Caucasian	14.2 ± 3.2		Mean
Englhard et al, 2016 23	CT	Mixed (Caucasian, Asian)	18.3 ± 3.1		Mean
Englhard et al, 2016 23	Endoscopy	Mixed (Caucasian, Asian)	18.8 ± 6.9		Mean
Chen et al, 2023 20	CT	Chinese	19.65 ± 3.82	20.48 ± 2.99	Bilateral
Kim et al, 2024 44	CT	Korean	18.73 ± 7.52		Mean
Poetker et al, 2004 22	CT	Caucasian	11.4 ± 2.6		Nasal base view
Hosseini et al, 2020 21	CT	Caucasian	10.77 ± 6.02		Mean
Ichimura and Ishizuka, 1997 45	Endoscopy	Japanese	28.9 ± 6.3		Mean
Jasso-Ramírez et al, 2018 46	Endoscopy	Hispanic	25.07 ± 5.0	24.07 ± 4.8	Bilateral
San Nicoló et al, 2020 24	MRI	Caucasian	28.6		Range: 12.7 to 39.3 degrees

Abbreviations: CT, computed tomography; MRI, magnetic resonance imaging.

Values are presented as mean ± standard deviation, or range.

a Measured at Sellion.

From a surgical perspective, these anatomical insights have direct clinical implications. Management of thick nasal envelopes is best approached in a staged manner that begins preoperatively, with dermatologic optimization to improve skin quality, reduce inflammation, and control sebaceous activity. Topical regimens such as retinoids, alpha hydroxy acids, and salicylic acid have been shown to reduce edema and enhance healing outcomes. 2 11 26 Intraoperatively, conservative handling of soft tissue is essential. Defatting techniques have limited long-term efficacy 5 ; meticulous sub-SMAS thinning with preservation of the vascular plexus is therefore preferred. Adjunctive use of platelet-rich fibrin (PRF) further supports wound healing and edema control. Gode et al demonstrated that supratip thickness remained consistently lower in the PRF group compared to controls up to 3 months postoperatively, suggesting sustained benefit. 27 28 Nevertheless, addressing the STE alone is insufficient. Reinforcement of the cartilaginous framework is often required to maintain projection and definition under thick envelopes. 29 30 Our septal measurements confirm sufficient robustness of the caudal septum (mean 2.09 ± 0.30 mm) for graft harvest, supporting the use of septal extension grafts, columellar struts, and lateral crural strut grafts in patients with thick envelopes. 11 29 30 Because Asian and African American patients often present with smaller quadrangular cartilage, 4 18 preoperative ultrasonography is particularly valuable for anticipating when septal cartilage alone may be insufficient. In such cases, planning for alternative donor sites such as auricular cartilage, the septal cartilage–bone complex, or costal grafts can reduce intraoperative uncertainty and strengthen the process of informed consent. 18 In addition, selective suturing (e.g., transdomal, interdomal) can augment tip stability but rarely suffices alone; durable results are best achieved when combined with robust structural grafting. 8 14 16 The functional aspect of rhinoplasty is equally critical and should be integrated alongside aesthetic planning. Preoperative ultrasonography of the internal nasal valve provides objective functional insight and allows the identification of patients at increased risk of obstruction. It also facilitates surgical planning by guiding the use of spreader grafts or flaring sutures. This integration of aesthetic and functional planning ensures that surgical refinements enhance both nasal appearance and function.

Septal imaging is technically challenging because the ultrasound beam often runs nearly parallel to the reflective cartilage surface, and vestibular air attenuates acoustic transmission, obscuring deeper margins. 31 Several refinements can mitigate these limitations. Liberal application of thick coupling gel or the use of a standoff pad improves near-field coupling; shallow imaging depth and a high-frequency, small-footprint transducer (e.g., 15–18 MHz “hockey stick”) enhance boundary definition; and oblique insonation helps reduce angle-dependent artefacts. 5 7 31 Transducer pressure should be kept minimal during thickness measurements to avoid compression-related bias of superficial tissues. In addition, rotating the transducer laterally and applying gentle pressure against the lateral nasal wall can collapse the vestibule, expel residual air, improve gel–mucosa contact, and facilitate more continuous visualization along the septum. 31 Collectively, these refinements reduce air–mucosa artefacts and improve the reliability of septal morphometry in rhinoplasty planning.

Beyond preoperative planning, ultrasonography offers a non-invasive, radiation-free method to longitudinally monitor changes in the nasal framework. This is particularly valuable in cases involving autologous cartilage grafts, where surgeons can objectively track the stability of the graft and detect potential postoperative cartilage resorption. Furthermore, given the risk of alar collapse or nasal valve stenosis, especially in complex revision rhinoplasty, serial ultrasound can assess valve patency and the integrity of the cartilage framework. This supports postoperative decision-making and planning of any subsequent interventions.

This study has several limitations, including a relatively small sample size. Ultrasonographic measurements were not validated against CT or intraoperative findings, and interobserver reproducibility was not assessed, which limits the generalizability of our results. In addition, functional correlation between INVA and airflow or patient-reported outcomes was not included, restricting interpretation of its clinical relevance. Future studies should validate ultrasonographic measurements against established standards, test reproducibility across operators, and explore longitudinal associations with both aesthetic and functional outcomes.

### Conclusion

Ultrasonographic assessment offers significant benefits in preoperative rhinoplasty planning, particularly for patients with thick nasal skin. The objective, quantitative measurements provided by ultrasound enhance surgical precision and predictability, potentially improving both aesthetic and functional outcomes.
